# Artificial Intelligence Sensing: Effective Flavor Blueprinting of Tea Infusions for a Quality Control Perspective

**DOI:** 10.3390/molecules29030565

**Published:** 2024-01-23

**Authors:** Andrea Caratti, Angelica Fina, Fulvia Trapani, Carlo Bicchi, Erica Liberto, Chiara Cordero, Federico Magagna

**Affiliations:** Dipartimento di Scienza a Tecnologia del Farmaco, Università degli Studi di Torino, 10125 Turin, Italy; andrea.caratti@unito.it (A.C.); angelica.fina@unito.it (A.F.); fulvia.trapani@unito.it (F.T.); carlo.bicchi@unito.it (C.B.); erica.liberto@unito.it (E.L.); federico.magagna@gmail.com (F.M.)

**Keywords:** flavor blueprint, black tea infusions, sensomics-based expert system, industrial quality control, artificial intelligence sensing, accurate flavor screening

## Abstract

Tea infusions are the most consumed beverages in the world after water; their pleasant yet peculiar flavor profile drives consumer choice and acceptance and becomes a fundamental benchmark for the industry. Any qualification method capable of objectifying the product’s sensory features effectively supports industrial quality control laboratories in guaranteeing high sample throughputs even without human panel intervention. The current study presents an integrated analytical strategy acting as an Artificial Intelligence decision tool for black tea infusion aroma and taste blueprinting. Key markers validated by sensomics are accurately quantified in a wide dynamic range of concentrations. Thirteen key aromas are quantitatively assessed by standard addition with in-solution solid-phase microextraction sampling followed by GC-MS. On the other hand, nineteen key taste and quality markers are quantified by external standard calibration and LC-UV/DAD. The large dynamic range of concentration for sensory markers is reflected in the selection of seven high-quality teas from different geographical areas (Ceylon, Darjeeling Testa Valley and Castleton, Assam, Yunnan, Azores, and Kenya). The strategy as a sensomics-based expert system predicts teas’ sensory features and acts as an AI smelling and taste machine suitable for quality controls.

## 1. Introduction

The tea plant has great economic importance and, after water, tea infusions are the most consumed beverages in the world; they are prepared by hot water extraction of dried leaves from *Camellia sinensis* L. Kuntze [1,2,3]. According to their manufacturing, teas can commercially be classified into three main categories: non-fermented (*white* and *green* teas), partially fermented (*oolong* and *paochong* teas), and fully fermented (*black* tea) [4,5]. Green and black teas are widespread in the world, while the other kinds of tea (white, yellow, and oolong) are mainly consumed in producing countries [6].

Tea is characterized by important physiological and potential health benefits [7,8,9,10] due to the presence of many bioactive chemical constituents. The chemical composition of the dried tea plant (green or black) includes primary metabolites such as proteins, free amino acids, carbohydrates, vitamins, minerals, and specialized metabolites, i.e., polyphenols, purine alkaloids (methyl xanthines and, above all, caffeine), chlorophyll, and other volatile and non-volatile compounds [1].

The unique combination of non-volatile solids extractable from tea leaves and the complex volatile fraction evokes tea’s characteristic flavor, which is fundamental in influencing its market value and consumer choices. It is well known that the quality of food is not only guaranteed by its safety and health properties but a significant role is played by its hedonic profile, especially flavor, and appearance [11,12,13,14,15,16,17,18,19,20].

Industrial qualification of black teas is mainly based on known quality markers belonging to the phenolic fraction of plant-specialized metabolites [1]. Available methods, some of them also normalized and standardized as ISO norms, are directed to the whole fraction (Folin–Ciocalteu test for the total polyphenol content) or specific metabolites (theaflavins and flavan-3-ols). Moreover, since the appreciation of tea infusions is strongly conditioned by flavor and appearance, quality control sometimes also includes sensory evaluation of model infusions performed by trained panels.

If the chemical characterization is intrinsically objective, being directed to target fractions/analytes and based on molecular methods that can be validated according to consensus guidelines, the sensory evaluation is more complicated, time-consuming, and intrinsically less objective. It could fail in the comparative evaluation of samples over wide time frames or for high-throughput testing. 

Tea flavor has represented an intriguing research topic since the 1930s [5], and this area of investigation is still active today. The increase in the industrial production of *ready-to-drink* beverages requires an in-depth knowledge of the quality of raw materials (traceability and flavor features) to obtain products with excellent yet benchmarked flavor.

The literature reports several studies focused on compounds responsible for the characteristic flavor of teas, differing by geographical provenience/botanical variety. In 2006, Schuh and Schieberle [21] applied the *molecular sensory science* approach, nowadays referred to as *sensomics*, to identify and quantify key aroma compounds in the infusion prepared from Darjeeling (India) black teas. The approach includes solvent-assisted flavor evaporation (SAFE) followed by gas chromatography–olfactometry (GC-O) with aroma extract dilution analysis (AEDA) to identify potent odorants and their odor qualities. With the stable isotope dilution assay (SIDA), the accurate quantitative determination of odorants highlights those exceeding the odor threshold (OT) in the sample, thereby indicating how to reconstruct an aroma recombinates that evokes the unique and distinctive aroma identity (*aroma blueprint*) of the product [22]. The authors identified a total of 42 impacting odorants both in the leaves and in the resulting infusions with some quantitative differences. Among 16 character impact odorants, terpene alcohols (*geraniol* and *linalool*), Strecker aldehydes (*2-methyl propanal*, *2-* and *3-methyl butanal*), and carotenoid derivates (*β-damascenone*) significantly increased their concentration after the infusion process. 

A complementary analytical procedure was adopted by Scharbert and co-workers [23] to identify the key molecules generating the taste perception after tea infusion consumption. Their studies on Darjeeling tea revealed that the main contributors to the astringent taste perceived are a series of compounds belonging to the flavonoids class, i.e., *flavan-3-ol glycosides*. Borse et al. [24] investigated the characteristic distribution of volatiles and non-volatiles in black teas originating from different regions of India (Darjeeling, Assam, Nilgiris, etc.). In that study, tea chemical fingerprints were identified by associating physicochemical assays, spectrophotometric, and liquid chromatography (LC) analyses for the determination of flavonoids and *caffeine*, while simultaneous distillation–extraction (SDE) followed by GC coupled to mass spectrometry (MS) was used to map the volatile fraction. In particular, 25 volatile flavor compounds were demonstrated to be diagnostic in discriminating Indian tea samples from the others.

In 2008, Wang and co-workers [25] profiled 56 teas with different degrees of fermentation (green, oolong, and black teas) using LC and head-space solid-phase micro-extraction (HS-SPME) followed by gas chromatography–mass spectrometry (GC-MS). They found that neither the total nor individual *catechin* content was different in green and oolong teas among the investigated samples, while the post-harvest process (fermentation) was responsible for major differences in volatile distribution. Within the volatiles, five components (*(E)-2-hexenal*, *benzaldehyde*, *6-methyl-5-hepten-2-one*, *methyl salicylate*, and *indole*) were able to discriminate unfermented and fermented teas, while *(E)-2-hexenal* and *methyl salicylate* together supplied an index to differentiate semi- and fully fermented teas. Special teas (e.g., Chinese green tea *Jingshan cha* and *Longjing*) were recently investigated for aroma blueprinting by sensomics [26,27], posing the basis for the application of a new concept/methodology termed sensomics-based expert system (SEBES) [28]. 

The methodological approach SEBES is able to “characterize key food odorants with one single analytical platform and without using the human olfactory system, that is, by artificial intelligence smelling” [28]. With SEBES, the set of key food odorants (KFOs) of a product can be accurately quantified and by combining quantitative data with OTs, the OAVs are automatically estimated and aroma features are predicted with great accuracy. This way, the food odor code is “defined without using the human olfactory system” [28]. This application of Artificial Intelligence (AI) concepts to food sensory properties is intriguing yet attractive for the industry’s quality control (QC), where primary materials and finished products have to be qualified and benchmarked against references. 

The AI prediction of aroma and taste (i.e., flavor blueprinting), together with quality related to bio-active compounds, is achieved by developing and validating an integrated SEBES analytical strategy suitable for quality control (QC) in industrial laboratory environments. To support the high throughput required by QC and quality assurance (QA) labs, flavor and quality markers are quantitatively profiled by fully automated GC-MS and LC with an ultra-violet diode array (UV/DAD), avoiding time-consuming sample preparation. In particular, for aroma blueprinting in solution (IS), SPME-GC-MS is implemented with Standard Addition (SA) quantification, while for taste blueprinting, LC-UV/DAD is with external standard (ES) calibration of key aromas and marker compounds [29].

The goal is to provide an industrial laboratory with an efficient and objective quality control strategy capable of answering many questions about tea quality (characterizing compounds) and sensory features (taste and aroma above all) while providing countable metrics for all incoming batches. Metrics can define similarity or dissimilarity against a benchmark quality or a company reference, facilitating decision-making and strategies [30].

## 2. Results and Discussion

The number of chemicals effectively contributing to the flavor of food (key aroma, key taste, and trigeminally active compounds) is relatively small. Molecular sensory science, nowadays referred to as sensomics, is the reference discipline to correlate food flavor perception with its molecular code [31]. Sensomics has established a protocol that combines multiple discrete steps aimed at isolating, extracting, and concentrating odor-active and taste-active compounds before their qualification (odor/taste characterization and description), accurate quantification, and validation in flavor recombinates. Once decrypted, the molecular flavor code is a valuable tool for predicting product sensory features [28,31]. However, the analytical approaches adopted by sensomic workflows are not compatible with, nor suitable for, QC and QA food assessment, where simpler platforms and full automation are key characteristics for high sample throughput and method reliability. 

With this perspective, the current study aims to develop and validate molecularly resolved analytical tools, compatible with QC and QA labs, for AI sensing and prediction of black tea infusion flavor and quality. The strategy should therefore integrate an accurate quantification of volatile key aromas and non-volatile taste-active and quality markers in a suitable range of concentrations covering real-world samples of industrial interest. 

Concerning aroma-active compounds, the adoption of a suitable sampling technique is fundamental to obtaining a meaningful picture of components evoking the sensory identity and quality profile of the product [32]. In this respect, so-called high-concentration-capacity sampling techniques (HCC) [3,33,34,35,36] are the elective route to achieving suitable selectivity, sensitivity, and quantification accuracy to provide high-throughput informative analysis in full automation. In a previous study, several HCC approaches were tested for their capability to efficiently and accurately delineate the aroma blueprint of black teas from Ceylon [3]. Of the tested techniques, HS-SPME-GC-MS was the most suitable for the in-solution sampling of key-aroma compounds, providing the possibility of accurate quantification of analytes by standard addition (SA) and calibration [37].

Regarding taste-active and trigeminally active compounds, teas are characterized by a complex pattern of specialized metabolites belonging to the flavonoid and methyl xanthine classes, which are responsible for taste and trigeminal sensations. In particular, *caffeine* is responsible for bitterness while glycosidic derivates of flavonols (*quercetin*, *myricetin*, and *kaempferol*) are the most taste-active analytes, imparting a mouth-drying and velvety-like astringency. In addition, an important role in defining tea quality (dried plant and related infusion) is also played by flavan-3-ols, which exert a minor but not negligible contribution on overall tea astringency perception. Depending on their concentration, flavan-3-ols (catechins) and dimeric derivates (theaflavins) evoke a puckering astringency and rough oral sensation [38].

The following sections present and critically discuss the experimental results on the quali-quantitative profiling of the most potent flavor components of selected teas after standardized infusion according to the EMA/HMPC/283630/2012 Committee on Herbal Medicinal Products (HMPC) protocol. Teas were all fermented (i.e., black teas) and were from Ceylon (*Flowery Orange Pekoe*—FOP), India (Assam, Darjeeling Testa Valley, Darjeeling Castleton), Portugal (Azores), China (Yunnan), and Kenya.

### 2.1. Tea infusion Volatile Profiling by IS-SPME-GC-MS

As a first step, GC-MS analyses acquired in full-scan mode enabled a comprehensive mapping of the volatile fraction of tea infusions. Volatiles were reliably identified by their EI-MS fragmentation patterns, compared to those present in commercial libraries (Wiley [39] and NIST 2014 [40]). The acceptability criteria for putative identification were direct match factor (DMF) > 900 and linear *I^T^_s_* tolerance ± 5 units. When available, pure reference compound confirmation was performed. Table 1 reports a list of 44 volatile marker compounds together with their retention times, experimental *I^T^_S_*, odor descriptors, and presence in analyzed samples. Although the number of detected and reliably identified volatiles was lower compared to the GC×GC methodology [2,3] adopted in previous investigations, the proposed strategy with IS-SPME-GC-MS allowed for efficient mapping of the most relevant markers carrying information about tea aroma profile, plant origin, and manufacturing practices.

Results on the volatile profiling showed a different distribution of analytes within the selected samples; Ceylon and Darjeeling Testa Valley (India) were characterized by a more complex volatile fraction, showing a matching of 42 detected analytes over 44 targets. The same outcome could not be observed with the other teas from Darjeeling (Castleton), where only 26 volatiles were detected, demonstrating how different tea gardens located in the same country lead to distinctive products. On the other hand, tea samples from Azores (Portugal) and Yunnan (China) showed a less complex fraction of volatiles, also in terms of key aroma compound distribution; indeed, within all identified analytes, only 15 compounds were mapped in Azores tea and 23 in Yunnan tea.

Qualitative profiling results show that some volatiles are ubiquitous in all tea infusions, although quantitative differences deserve some comments. Of those detected in all samples, Strecker aldehydes (*2-methyl butanal* and *3-methyl butanal*) and volatile terpenes (*linalool* and its related *3,6-oxides*, and *geraniol*) are the most relevant in the aroma definition. Phenyl propanoid derivates, a group of characteristic components in tea, followed a slightly different behavior; *phenylacetaldehyde* and *benzaldehyde* were detected in all samples, while *benzyl alcohol* and *2-phenyl alcohol* were present only in some products (both for Ceylon and Darjeeling TV, *benzyl alcohol* in Assam tea, the others in tea from Kenya). Within the entire set of volatiles, an important role is played by saturated and unsaturated aldehydes, which originate from the oxidation of fatty acids and contribute to defining the aroma of tea infusions. In this case, it is interesting to point out that some short-chain linear aldehydes (*hexanal*, *heptanal*) are present in all infusions, while unsaturated C7-C10 aldehydes have characteristic patterns in Ceylon, Assam, Darjeeling TV, and Kenya samples. 

The next step focused on key aroma quantitation by Standard Addition (SA), a well-established internal calibration approach suitable when the so-called matrix effect cannot be neglected and likely has an impact on method accuracy. Indeed, in the case of tea infusions, the release/behavior of volatiles is strictly influenced by their interaction with non-volatile components (e.g., polyphenols, alkaloids, organic acids, pigments). The next section introduces the SA procedure and quantitation results.

### 2.2. Key Aroma Marker Quantitation by Standard Addition (SA) and IS-SPME-GC-MS

The standard addition procedure, widely used as a quantitation approach, consists of a series of experiments in which the original sample and a suitable number (at least four concentration levels) of aliquots of the sample, spiked with increasing and known amounts of reference compounds, are submitted to the analytical process.

When using the single addition method, the analyte concentration in the sample can be estimated from Equation (1):

(1) $$A_{\left(0+a\right)}=\frac{A_{0}}{W_{0}}W_{a}+A_{0}$$

where *W*_0_ is the amount of analyte in the matrix, *W_a_* is the amount of analyte added to the sample, *A*_0_ is the instrumental response obtained from the analysis of the original sample, and *A*_(0+*a*)_ is the instrumental response of the analyte obtained from the analysis of the spiked sample.

A preferable and more accurate procedure, which was applied in this study, includes multiple standard additions. With multiple SAs, a linear regression analysis evaluates the terms *W_a_* and *A*_(0+*a*)_ so the amount of analyte in the matrix (*W*_0_) is given by the ratio between the intercept and the slope, Equation (2):

(2) $$\frac{b}{a}=\frac{A_{0}}{\frac{A_{0}}{W_{0}}}$$

Standard addition is a quantitation approach that can be carried out in different ways: (*a*) by spiking the target analyte(s), in a gaseous state, into the sample headspace (gas phase addition—GPA); (*b*) by spiking the analyte(s), dissolved in a suitable solvent, directly onto the sample (sample phase addition—SPA); or (*c*) by spiking the stable-isotope-labeled analyte(s) dissolved in a suitable solvent (stable isotope dilution analysis—SIDA) onto the sample. The present study adopted the SPA protocol, suitable for its ease of implementation and automation and its cost-effectiveness compared to isotopically labeled standards.

The analytical protocol, rationalized in the Experimental section, consisted of (*a*) three replicate infusions prepared from each tea sample and (*b*) three standard addition levels for each infusion (plus the analysis of the original sample). Acetone was selected as a solvent for spiking solutions because it guaranteed the full solubilization of all target analytes, being miscible with water. For each calibration step, two analytical replicates were acquired.

Thirteen key odorants were accurately quantified: 3-methyl butanal, 2-methyl butanal, hexanal, (*Z*)-4-heptenal, phenyl acetaldehyde, linalool, (*E,Z*)-2,6-Nonadienal, (*E*)-2-nonenal, (*E,E*)-2,4-nonedienal, geraniol, (*E,E*)-2,4-decadienal, β-damascenone, and β-ionone. They are listed in Supplementary Table S1 (ST1) together with chromatographic information on retention times (*t_R_* min), Target/Qualifier Ions *m*/*z*, calibration functions and determination coefficients (*R*^2^), and precision and accuracy results. For the method’s performance parameter evaluation, see the experimental section, Section 3.9.

Table 2 lists quantitation results, obtained using the SA calibration technique and IS-SPME-GC-MS (SIM) for key aromas in tea infusions. The results on Ceylon teas are reported as the mean value obtained from four commercial batches from the same harvest year. Data are expressed as µg/L in the infusion.

Experimental results are consistent with those obtained by Schuh and Schieberle [21], who adopted a more complex procedure for the isolation and accurate quantification of potent odorants, i.e., SIDA and solvent-assisted flavor evaporation (SAFE) followed by GC–olfactometry (GC-O) and aroma extract dilution assay (AEDA). 

**Figure 1:**
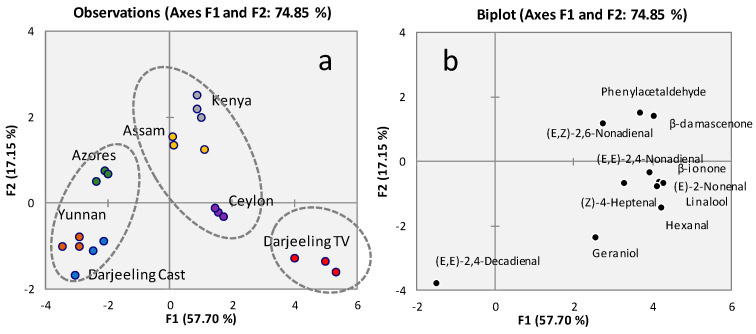
PCA results. (**a**) Score plot on the first and the second principal components (F1 F2 plane) based on key aroma compound quantitative data determined in infusions prepared from tea leaves (Table 2). For Ceylon teas, an average profile obtained from four lots was considered; (**b**) loadings plot with the distribution of quantified analytes.

An unsupervised multivariate approach (i.e., Principal Component Analysis—PCA) provides prompt information on samples’ natural clustering based on compositional similarities. Figure 1a shows the score plot on the first and the second principal components (F1-F2 plane) obtained by analyzing the distribution of targeted odorants in all sample replicates. The first principal component (F1) explains 57.70% of the total variance (74.85%), contributing most to the discrimination of samples, while the second principal component (F2) has a minor informative influence (17.15%). Three main groupings (ellipses with dotted lines) arbitrarily delineated by the authors can be observed in the score plot of Figure 1a: from left to right with increasing F1 score values, the Darjeeling Castleton, Azores, and Yunnan, followed by Assam, Ceylon, and Kenya, and finally Darjeeling Testa Valley (TV) with higher score values along F1. The distribution of variables as a function of the first two principal components is reported in the loadings plot of Figure 1b. Analytes providing the most information on the F1 axis, and likely more abundant in related infusions, directly correlate with Ceylon, Assam, Kenya, and Darjeeling TV teas. 

Interestingly, Darjeeling teas, although produced in the same region of India, showed distinctive yet different aroma-active compound patterns, with Darjeeling TV characterized by higher amounts of these analytes. On the other hand, teas from Ceylon, Assam, and Kenya were clustered together, suggesting similar aroma features.

Experimental results indicate that the most potent odorants characterizing these kinds of tea are present in a wide range of concentrations, to be specific, 0–4 µg/L for *(Z)-4-heptenal*, *(E,Z)-2,6-nonadienal*, *(E)-2-nonenal*, *(E,E)-2,4-nonadienal*, *(E,E)-2,4-decadienal*, *β-damascenone,* and *β-ionone*, while *hexanal*, *linalool*, *geraniol*, *phenyl acetaldehyde*, and *2* and *3-methyl butanal* are in the range 0–100 µg/L.

Darjeeling TV has a peculiar profile described by the flowery terpenes *linalool* (54.48 µg/L) and *geraniol* (24.83 µg/L), the green-grassy note from *hexanal* (63.49 µg/L), and fatty nuances likely modulated by unsaturated aldehydes *(E)-2-nonenal* and *(E,E)-2,4-nonadienal*. The commercial selection of Ceylon infusions are described by higher amounts of *(Z)-4-heptenal* (0.98 µg/L) and *(E,Z)-2,6-nonadienal* (0.56 µg/L). Other key volatiles such as *phenylacetaldehyde*, *β-damascenone,* and *β-ionone* play a role in defining the peculiar profiles of Ceylon, Assam, Kenya, and Darjeeling TV.

Teas from Kenya and Assam are characterized by higher amounts of Strecker aldehydes *3-methyl butanal* (49.12 µg/L for Assam and 84.89 µg/L for Kenya) and *2-methyl butanal* (63.12 µg/L for Assam and 89.97 µg/L). Moreover, within the cluster of Ceylon, Assam, and Kenya, other major differences occur for *β-ionone*, more abundant in Ceylon (2.16 µg/L) and Kenya (1.92 µg/L).

In general, Azores, Yunnan, and Darjeeling Castleton showed a weaker profile of aroma-active analytes; in particular, many key odorants (unsaturated aldehydes and the nor-isoprenoids *β-damascenone* and *β-ionone*) were not detected and quantified in the related infusions. However, tea from the Azores showed a fairly high amount of Strecker aldehydes (*3-methyl butanal*, *2-methyl butanal* 46.69 µg/L), which likely imparts malty notes.

Nevertheless, the great variability in the quantitative distribution of key odorants does not necessarily lead to a meaningful characterization of the selected samples for their sensory quality. Odorants, besides their intrinsic potency that is related to the binding with odor receptors (ORs), should be effectively released by the food matrix to reach the olfactory epithelium and trigger retronasal olfaction (i.e., aroma perception). The ratio between the analyte’s concentration in the sample (i.e., tea infusion) and its odor threshold (OT, i.e., the lowest concentration of a compound that is just enough for the recognition of its odor [38]) in water provides a more realistic perspective of the overall aroma quality and odorant balancing. For this reason, the contribution of the analytes in the prediction of samples’ sensory profiles is evaluated by applying the SEBES concept.

### 2.3. Aroma Blueprinting by AI Smelling Based on Sensomics

As reported by Schieberle and co-workers in their studies on Darjeeling black tea (infusions and dried leaves) [21,38], a group of 24 odorants with high flavor dilution (FD) factors was recognized to play a prominent role in defining the characteristic aroma of the final infusion. Within these 24 chemicals, 16 were identified as having a high odor activity value (OAV), i.e., the ratio between the odorant concentration in the food vs. its odor threshold. It is commonly assumed that the higher the OAV value, the higher its contribution to the overall sensory perception.

Among the most odor-active compounds revealed by sensomics (16), i.e., those with OAV values ≥ 1 (value recognized to be significant in contributing to the flavor of food [44]), the current method reliably monitors 13 of them with a fully automatized procedure that avoids laborious sample-preparation steps. With IS-SPME-GC-MS, an effective aroma blueprinting method is achieved that supports, or even replaces, sensory panel evaluation in the perspective of quality benchmarking and quality controls. 

Regarding aroma features, the Strecker aldehydes formed during the fermentation process [1] (*2-methyl butanal* and *3-methyl butanal*) concur to an intense malty perception, while *phenylacetaldehyde* evokes a pleasant honey-like note. Responsible for floral notes are *geraniol* (rose-like) and *β-ionone* (violet-like), and for fruity notes, *linalool* and *β-damascenone*. An important contribution to the black tea aroma identity is also provided by the green and grassy notes of *hexanal*, *(E)-2-nonenal*, and *(E,E)-2,4-nonadienal*. Fatty perception is modulated by C9 unsaturated aldehydes, while fishy notes are from [*(Z)-4-heptenal*] and fatty/fried by [*(E,E)-2,4-decadienal*]. All these aldehydes are formed from the enzyme-catalyzed oxidation of fatty acids during plant growth and manufacturing processes [42].

**Figure 2:**
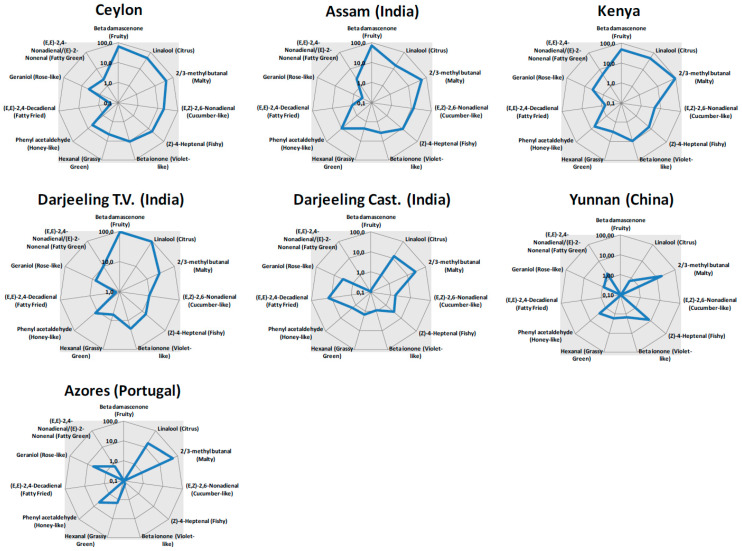
Spider diagrams with Odor Activity Values (OAVs) illustrating the contribution of each key aroma compound to the aroma perception of selected teas. OAV data are reported on logarithmic scale. Ceylon tea’s aroma profile is reported as average value calculated from four different lots.

The aroma blueprint of the infusions is visualized as spider diagrams based on OAV values in a logarithmic scale (Figure 2). By this visualization for each compound, the concentration is associated with the relative OT in water and the odor descriptor. The spider diagrams show that most tea samples have a specific aroma profile characterized by a good balance of markers; in general, teas from Ceylon, Assam, and Kenya likely have a similar aroma profile, especially for some notes such as fruity (*β-damascenone*), malty (Strecker aldehydes), honey-like (*phenyl acetaldehyde*), and cucumber-like (*(E,Z)-2,6-nonadienal*). On the other hand, Assam infusion is characterized by a lower content of terpene derivates (*geraniol* and *linalool*) than the other teas, likely resulting in weaker citrus and rose-like notes. The aroma profile confirms the great diversity of samples from Darjeeling.

A very important outcome deriving from the calculation of OAVs consists of the explanation of the role played by each specific analyte in the definition of the aroma profile. Although present at very low concentrations (range 0–4 µg/L), some analytes such as *β-damascenone*, *β-ionone*, *(Z)-4-heptenal*, and *(E,Z)-2,6-nonadienal* are extremely potent, being characterized by very low OTs (e.g., 0.004 µg/L for *β-damascenone*); as an example, *β-damascenone* generates the maximum OAV of 94.8 in Darjeeling Testa Valley tea, where OAVs were higher also for other key volatiles (i.e., *linalool*, *3-methyl butanal*, and *2-methyl butanal*). On the contrary, despite their high concentration in the beverage, *hexanal,* and *phenylacetaldehyde* likely play a minor role in the definition of tea aroma, being less active than odorants (higher OTs, 10 and 6.3 µg/L, respectively).

### 2.4. Taste-Active Compounds’ and Quality Markers’ Accurate Quantitative Profiling by LC-UV/DAD

Tea infusions were then profiled by LC-UV/DAD to quantitatively map non-volatiles responsible for taste and trigeminal perception and for quality features [8,29]. The analytical method was optimized and verified for the accurate quantification of 19 informative chemicals in a single analytical run. Analytes are listed in Table 3, together with the taste threshold (TT) and average amounts (mg/L) from three replicate infusions for each sample. The analytical method’s figures of merit, including precision and accuracy, are discussed in the Experimental section and detailed in Supplementary Table S1 (ST1).

**Figure 3:**
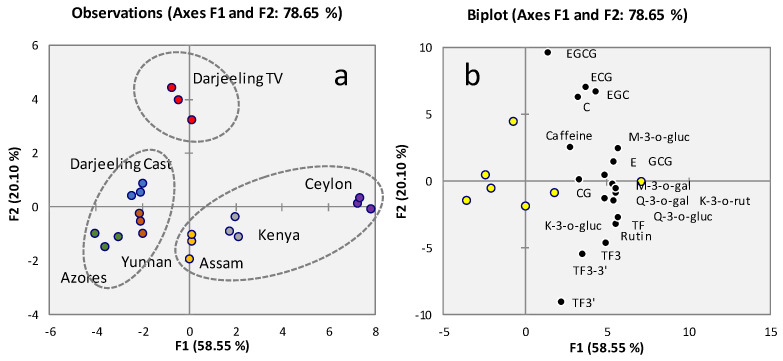
PCA results. (**a**) Score plot of the first and the second principal components (F1 F2 plane) based on key taste and quality compounds’ quantitative data determined in infusions prepared from tea leaves (Table 3). For Ceylon teas, an average profile obtained from four lots was considered; (**b**) loadings plot with the distribution of quantified analytes and centroids (yellow circles) for analyzed teas.

Principal Component Analysis was applied to the data matrix (quantitative results) of non-volatiles to evaluate the presence of natural clusters within selected samples. Figure 3a shows the score plot of the first and the second principal components (F1-F2 plane); of note, the variance explained by the first two components is quite high and similar to that resulting from key aroma patterns shown in Figure 1a (58.55% F1, 20.10% F2, for a total variance of 78.65%). Moreover, sample clusters based on taste and quality marker distribution are similar to those shown in Figure 1a generated by volatile patterns. Darjeeling Testa Valley is characterized by a fingerprint that drives its independent clustering; it is described by a peculiar pattern of *flavan-3-ols* (Figure 3b) such as *epigallocatechingallate* (EGCG), *epigallocatechin* (EGC), *catechin* (C), and *epicatechingallate* (ECG). Experimental results on Darjeeling black tea are consistent with those obtained by Scharbert and co-workers in 2004 [23]. Ceylon, Assam, and Kenya teas’ sub-classification is mostly driven by theaflavins and *flavonol-3-o-glycosides*. On the other hand, tea samples from the Azores, Yunnan, and Darjeeling Castleton inversely correlate with these variables (weaker profiles).

The quantitative distribution of monitored markers will have an impact on teas taste and trigeminal perception. Flavan-3-ols, originally present in tea leaves, can undergo substantial changes during post-harvest treatments (oxidative phenomena during fermentation), leading to the formation of high-molecular-weight dimeric (theaflavins) and oligomeric (thearubigins) derivates. For this reason, in fermented black tea, quantitative differences in flavan-3-ols and theaflavins can be ascribed to both the geographical origin and the technological processing. Conversely, the levels of flavonols and *caffeine* remain unchanged during tea manufacturing, and thus, their variations are mostly influenced by the origin [3,42].

The high amount of flavan-3-ols but the low content of theaflavins in the Darjeeling Testa Valley infusion suggests lower oxidation during post-harvest treatment, which preserves the original amount of these analytes. On the other hand, teas from Ceylon, Assam, and Kenya, due to the higher levels of dimeric analytes, are likely more fermented. In addition, theaflavins are responsible for the characteristic dark orange-red color of black tea infusions, a piece of evidence that was confirmed by a visual inspection of the infusions. Indeed, teas from Darjeeling had a light yellow color due to the low concentration of these markers, in contrast with Ceylon tea. Tea from the Azores was confirmed to be characterized by a poor non-volatile fraction for all compounds, suggesting a low astringency capacity.

*Caffeine* is present in similar concentrations in all samples (230–260 mg/L), with the only exception of Azores tea (143 mg/L); this outcome is probably related to the standardization of the infusion process (incomplete extraction), which does not allow its exhaustive extraction from the matrix.

### 2.5. Taste Blueprinting by AI Tasting Based on Sensomics

In general, it can be assumed that the infusions with higher concentrations of flavan-3-ols and flavonol-3-o-glycosides (Ceylon and Darjeeling Testa Valley) are described by a more intense astringent sensation; however, to objectify the real contribution on the overall taste perception of the seven flavan-3-ols and the seven flavonol-3-o-glycosides, beyond the other markers, concentration data were associated with taste thresholds (Table 3).

**Figure 4:**
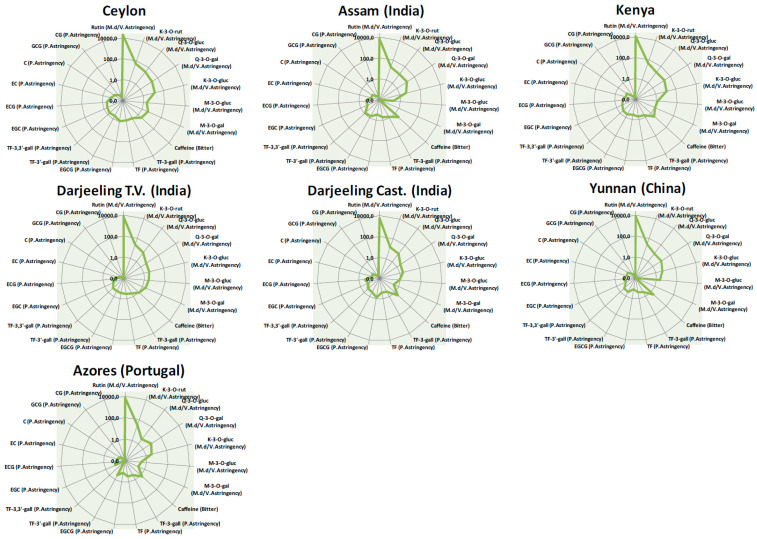
Spider diagrams with dose-over-threshold values (DoTs) illustrate the contribution of each key taste and quality marker to the sensory perception of selected teas. DoT values are reported on a logarithmic scale. Ceylon tea taste profile is reported as average values derived from four different lots.

Figure 4 visualizes, as spider diagrams, the dose-over-threshold (DoT, ratio of the concentration of each compound vs. its taste threshold) values, i.e., the taste blueprint profile of tea infusions. Key tastants are associated with their taste descriptors, e.g., bitter, p. (puckering) astringency, m.d/v (mouth-drying/velvety) astringency.

Within the entire class of monitored polyphenols, flavonol-3-ol glycosides are the most relevant for the definition of the taste blueprint, evoking a strong mouth-drying and velvety-like sensation [23,45]. In particular, *quercetin-3-o-rutinoside*, known as *rutin*, is the most representative marker, followed by *kaempferol-3-o-rutinoside* and the other derivates of *quercetin* (glucoside and galactoside). *Rutin* likely has a strong influence on the taste profile (upper part of the spider diagrams) since it is characterized by DoT values of three orders of magnitude higher than those of flavan-3-ols (very low taste thresholds, e.g., from 0.0009 mg/L for *rutin* to 0.30 mg/L for the glyosidic compounds *quercetin* and *kaempferol*). In addition, an important, although not primary, role for taste perception is also played by flavan-3-ols, especially those esterified with gallic acid; *epigallocatechingallate* (EGCG) is the most abundant *catechin* present in black teas (85.35 mg/L in Ceylon infusion, 177.23 mg/L in Darjeeling Testa Valley tea) [14,21]. The bitterness of the beverage is mainly related to the high content of caffeine, which, however, shows a low DoT value, being characterized by high TT.

As a final consideration, the higher the concentration of flavonoidic markers, the more intense the astringency sensation after tea consumption; this outcome can be expected for tea samples from Ceylon, Darjeeling TV, Assam, and Kenya.

## 3. Materials and Methods

### 3.1. Reference Compounds and Solvents

Pure reference compounds for odorant and key aroma identity confirmation and quantitation ((*E*)-2-nonenal, (*E,E*)-2,4-nonadienal, (*E,E*)-2,4-decadienal, 3-methyl butanal, 2-methyl butanal, hexanal, phenyl acetaldehyde, (*Z*)-4-heptenal, linalool, (*E,Z*)-2,6-nonadienal, β-damascenone, β-ionone, geraniol, phenyl acetaldehyde) and n-alkanes (n-C7 to n-C25) for (*I^T^_s_*) determination were from Merck (Milan, Italy).

Pure reference compounds for the quantitative determination of key tastants and quality markers ((−)-epigallocatechin, (+)-catechin, (−)-epigallocatechingallate, (−)-epicatechin, (−)-gallocatechingallate, (−)-epicatechingallate, (−)-catechingallate, theaflavin, theaflavin-3-gallate, theaflavin-3′-gallate, and theaflavin-3,3′-digallate) were supplied by Merck. 

Tea extract from black tea (≥80% theaflavin and theaflavin gallate basis), used as a reference for the theaflavin external standard calibration, was purchased from Fluka Analytical. Reference standard compounds of *myricetin-3-O-glucoside*, *myricetin-3-O-galactoside, quercetin-3-O-rutinoside* (rutin), *quercetin-3-O-galactoside, quercetin-3-O-glucoside, kaempferol-3-O-rutinoside, kaempferol-3-O-glucoside*, and *caffeine* were supplied by Extrasynthese (Lyon, France).

Internal standardization (ISTD) for volatile compounds was conducted with *1,4-dibromobenzene* from Merck. 

Solvents were all LC grade, from Merck: *acetone* (purity 99.5%), *acetonitrile* ACN (purity 99.9%), and *methanol* (purity 99.9%). *Water* used to prepare stock solutions, tea infusions, and LC mobile phases was obtained with a Milli-Q RG system (Millipore, Molsheim, France) in agreement with the ISO 9002 Quality Systems Standards.

### 3.2. Reference Solutions and Calibration Mixtures

Standard stock solutions at 10 mg/mL, containing pure aroma reference compounds and ISTDs, were prepared in acetone and stored in sealed vials at −18 °C for two weeks maximum. Standard spiking mixtures, adopted for quantification (Standard Addition SA procedure), were prepared by diluting standard stock solutions of each compound to different final concentrations in the range 5–500 ng/µL (5, 10, 25, 50, 100, 250, 500 ng/µL). The ISTF 1,4-dibromobenzene was added to all calibration solutions at 120 ng/µL.

For taste compounds, standard stock solutions were prepared in a water/ACN (9:1 *v*/*v*) mixture or methanol at a concentration of 1 mg/mL. Standard stock solutions were sealed and stored at −18 °C for two weeks. Standard calibration solutions at 100, 50, 25, 10, 5, 2.5, and 1 ng/µL were prepared by diluting suitable amounts of standard stock solutions in the same solvent (water for catechins and theaflavins, methanol for flavonols) and stored at −18 °C until analyzed.

### 3.3. Tea Infusions: Samples and Preparation

Four different lots of fermented dried tea leaves of homogeneous particle size from Ceylon (*Flowery Orange Pekoe*—FOP) were kindly supplied by Soremartec Italia Srl (Alba-Cuneo, Italy). Tea samples from India (Assam, Darjeeling Testa Valley, Darjeeling Castleton) were all graded as *Golden Flowery Orange Pekoe*—GFOP, but for those from Portugal (Azores), China (Yunnan), and Kenya, the OP grading was not available. Excluding the Ceylon teas, all the others were bought from a specialized tea shop in Turin (Italy).

Infusions were prepared by following the EMA/HMPC/283630/2012 Committee on Herbal Medicinal Products (HMPC) protocol: 3.0 g of dried leaves were suspended in 300 mL of ultrapure boiling water for 90 s and then filtered. Infusions were left to reach ambient temperature in closed glass flasks and directly analyzed by IS-SPME followed by GC-MS for aroma blueprinting. For taste and quality marker quantitative profiling, infusions were filtered through 0.45 mm and 25 mm nylon membrane syringe filters (Agilent, Little Falls, DE, USA) and then analyzed by LC-UV/DAD.

### 3.4. Automated in-Solution Solid-Phase Microextraction: Devices and Sampling Conditions

SPME sampling devices and fibers were purchased from Merck Supelco (Bellefonte, PA, USA). A Divinylbenzene/Carboxen/Polydimethylsiloxane (DVB/CAR/PDMS) *d_f_* 50/30 μm, 2 cm long fiber was chosen and conditioned before use, as recommended by the manufacturer.

Sampling conditions were set as follows: 20 mL of tea infusions were submitted to in-solution (IS) sampling for the quantitative profiling of key aroma compounds. In particular, for SA quantitation, 20 mL of tea infusion was sealed in a 20 mL headspace vial and spiked with suitable volumes of standard spiking solutions for each calibration level. Sampling was performed by exposing the SPME device to the tea infusion for 40 min at 50 °C.

### 3.5. Automated IS-SPME-GC-MS Instrumental Set-Up and Analysis Conditions

Automated SPME for IS sampling was performed using a MPS-2 multipurpose sampler (Gerstel, Mülheim a/d Ruhr, Germany) online integrated with an Agilent 7890 GC unit coupled to an Agilent 5977C MS spectrometer equipped with a High-Efficiency Source (HES) (Agilent, Little Falls, DE, USA) operating in EI mode at 70 eV. The transfer line was set to 280 °C. A HES Tune was used and the scan range was set to *m*/*z* 35–350 (full scan acquisition), with a scanning rate of 1000 amu/s, to obtain a suitable number of data points for each chromatographic peak to ensure reliable identification. Analyses were also acquired in Single-Ion Monitoring (SIM) mode for the accurate quantification of selected (key aroma) markers. A SE52 capillary column (95% polydimethylsiloxane, 5% phenyl—30 m × 0.25 mm *d_c_* × 0.25 µm *d_f_*) from Mega (Legnano, Milan, Italy) was used.

For Linear Retention Index (*I^T^*) determination, the *n*-alkane liquid sample solution (100 mg/L) was injected using the MPS-2 multipurpose sampler (Gerstel) under the following conditions: injection mode: split, split ratio 1:50, injector temperature 250 °C, injection volume 1 μL.

The analytes sampled by IS-SPME were thermally desorbed from the fiber for 5 min, directly into the GC injector, under the following conditions: injection mode: split, split ratio 1:20, injector temperature 250 °C. The carrier gas was helium, with a constant flow rate of 1.0 mL/min. The temperature program was 40 °C (1 min) to 170 °C at 3 °C/min and to 260 °C at 10 °C/min (5 min).

### 3.6. LC-UV/DAD Instrumental Set-Up and Analysis Conditions

LC-UV/DAD analyses were carried out on a Spectra System (SCM1000, P4000, AS3000) provided with a Spectra System UV6000LP Diode Array Detector (Thermo Fisher Scientific, Waltham, MA, USA). The LC column was a Supelco Ascentis^®^ Express RP-C18 (150 mm × 4.6 mm; spherical particles with fused core^®^ technology, 90 Å, 2.7 µm) from Merck; a pre-column (5 mm × 4.6 mm; spherical particles with fused core^®^ technology, 90 Å, 2.7 µm) was installed to preserve the analytical column for pollution.

Operative conditions were as follows: injection volume 8 µL; detection wavelengths: 280 nm for flavan-3-ols and caffeine, 350 nm for flavonol-3-O-glycosides, 380 nm for theaflavins; mobile phases: (A) H_2_O + 0.1% formic acid, (B) ACN + 0.1% formic acid; flow rate: 1.10 mL/min; mobile phase program: from 95% H_2_O (4.7 min) to 85% H_2_O (15.8 min) to 75% H_2_O (9.2 min), hold for 12 min, then to 65% H_2_O, and then to 100% ACN, hold for 3 min. Before re-injection, the LC system was stabilized for at least 5 min.

### 3.7. Data Acquisition and Data Processing

GC-MS data were acquired using a MassHunter WorkStation (Agilent Technologies, Wilmington, DE, USA), while LC analysis data acquisition and data handling were performed with ChromQuest 2.51 software (Thermo Fisher Scientific, Waltham, MA, USA). Data mining was conducted with XLSTAT 2014 (Addinsoft-New York, New York, NY, USA).

### 3.8. Method Validation Parameters

Method validation was designed according to Eurachem Guidelines [46] and performance quality evaluation was based on reference parameters of Commission Implementing Regulation (EU) 2021/808 of 22 March 2021 for quantitative methods in food applications [47]. Infusions prepared from Ceylon tea (named quality controls, QCs) were analyzed by IS-SPME-GC-MS and LC-UV/DAD and quantitative results collected over a period of six weeks were used for repeatability (intra-week) and intermediate precision (inter-week) assessments. Results on precision based on chromatographic responses (normalized over the internal standard for GC-MS and absolute areas for LC-UV/DAD) are reported in Supplementary Table S1 (ST1), expressed as percent coefficient of variation (CV%) together with chromatographic information on retention times (*t_R_* min), Target/Qualifier Ions (*m*/*z*) (GC-MS), detection wavelengths (nm) (LC-UV/DAD), calibration functions and determination coefficients (*R*^2^), and accuracy results from spiked QC infusions at two levels expressed as percent recovery (% Rec). Accuracy was tested at +10 and +25 µg/L for aroma compounds and +10 and +25 mg/L for tastants and quality markers.

### 3.9. Method Validation Results: Precision and Accuracy

For aroma compounds, results on quantified analytes referred to a fairly good precision with inter-week CV% values never exceeding 25% (results reported in Supplementary Table S1 (ST1)); this outcome was consistent with validation parameters included in the Commission Implementing Regulation (EU) 2021/808 in the case of analytes present at ppb levels (µg/L). Within aroma compounds, those with the best performances over the six-week validation period were *hexanal* (10.09%), *(E,Z)-2,6-nonadienal* (12.21%), *phenylacetaldehyde* (13.54%), and *geraniol* (14.25%). The most volatile odorants, i.e., Strecker aldehydes *3-methyl butanal* and *2-methyl butanal*, reported CV% values close to the acceptability threshold with 24.56 and 22.13%, respectively.

In the case of taste-active compounds, results concerning precision were all within the acceptable range established by the Commission Implementing Regulation (EU) 2021/808; CV% values were always below 18%. The higher value was obtained for flavan-3-ols with an inter-week CV% of 14.55% while a very good intermediate precision was obtained for theaflavin (5.16%), *caffeine* (6.0%), and flavonol derivates (10.60%).

Accuracy was determined by spiking suitable amounts of analytes within the working range. Percent recovery (% Rec) never exceeded the range ±20%, with better performances, as expected, for non-volatile compounds (average Rec % 103). 

## 4. Conclusions

The integrated analytical strategy proposed in this study enables the successful definition of the aroma and taste blueprint of black tea infusions according to the recently introduced concept of the sensomics-based expert system. The results show how the tuning of analytical methods with molecular resolution toward sensory-relevant analytes belonging to different chemical fractions (apolar volatiles/semi-volatiles and polar non-volatiles) makes a valuable yet functional characterization of samples based on their sensorial peculiarities possible. Targeted analytes were identified and validated by sensomics as key aroma, key taste, and trigeminally active compounds of black teas. Their accurate quantification and normalization over odor or taste thresholds provide diagnostic blueprints of the product’s sensory properties from which quality, origin, and thus economic values can be inferred. 

Since chemical blueprints are predictive of sensory properties, as an AI sensing tool, the current methodology supports industrial quality control laboratories with objective yet reliable differentiation and discrimination between teas of interest. The simultaneous quantification of 32 key analytes, in the absence of laborious sample preparation and with single chromatographic runs, generates a dataset ready for normalization over sensory thresholds that promptly projects into pictograms (i.e., the spider diagrams of Figure 2 and Figure 4) the predictable sensory features of a product, facilitating or even replacing the sensory qualification of large sets of incoming batches. The reliability of this approach is especially useful for QC and QA laboratories and, as with any confirmation method, it is suitable for accreditation under ISO 17025 [48] with great advantages for certification and competitiveness in the food industry.

## Figures and Tables

**Table 1:** List of the targeted volatiles detected in selected tea infusions by IS-SPME-GC-MS together with retention times (*t_R_* min), experimental *I^T^*, and odor descriptors as reported in reference literature [12,26,27,41,42]. Detected (d) and non-detected (nd) analytes are also reported for each tea sample; moreover, Ceylon tea is rendered as an average profile obtained from different quality grades. Analytes with an asterisk (*) are the key odorants confirmed by sensomics and subjected to accurate quantification in this study.

	Compound Name	*t_R_* min	*I^T^*_S_	Odor	Ceylon	Assam	Azores	Darjeeling Castleton	Darjeeling Testa Valley	Kenya	Yunnan
1	3-Methyl butanal *	2.61	541	Malty	d	d	d	d	d	d	d
2	2-Methyl butanal *	2.70	566	Malty	d	d	d	d	d	d	d
3	(*E*)-2-Pentenal	4.10	725	Green, apple	d	d	nd	nd	d	d	nd
4	(*Z*)-2-Penten-1-ol	4.85	788	-	d	d	nd	nd	d	nd	nd
5	Hexanal *	5.19	816	Grassy-green	d	d	d	d	d	d	d
6	(*E*)-2-Hexenal	6.82	856	Bitter, almond	d	d	d	d	d	d	d
7	1-Hexanol	7.65	873	Fruity	nd	nd	nd	nd	d	d	d
8	2-Heptanone	8.40	890	Sweet, fruity	d	d	d	nd	d	nd	nd
9	(*Z*)-4-Heptenal *	8.48	896	Fishy	d	d	nd	d	d	d	d
10	Heptanal	8.79	899	Oil, fatty	d	d	d	d	d	d	d
11	(*E*)-2-Heptenal	11.10	953	Fatty, almond-like	d	d	nd	nd	nd	d	nd
12	Benzaldehyde	11.10	954	Almond, burnt sugar	d	d	d	d	d	d	d
13	6-Methyl-5-hepten-2-one	12.42	984	Pungent, green	d	d	nd	nd	d	d	nd
14	(*E,Z*)-2,4-Heptadienal	12.79	993	Fatty, rancid	d	d	nd	d	d	d	nd
15	(*E,E*)-2,4-Heptadienal	13.43	1008	Fatty, rancid	d	d	nd	d	d	d	d
16	Limonene	14.17	1026	Citrus	d	nd	nd	d	d	d	d
17	2,2,6-Trimethyl cyclohexanone	14.51	1030	-	d	nd	nd	nd	nd	nd	nd
18	Benzyl alcohol	14.73	1035	Sweet, fruity	d	d	nd	nd	d	nd	nd
19	Phenyl acetaldehyde *	14.90	1039	Honey-like	d	d	d	d	d	d	d
20	(*E*)-2-Octenal	15.67	1055	Green, nut, fat	d	d	nd	nd	d	d	nd
21	Trans-linalool-3,6-oxide	16.98	1071	Sweet, floral, citrus	d	d	d	d	d	d	d
22	Cis-linalool-3,6-oxide	17.13	1087		d	d	d	d	d	d	d
23	Linalool *	17.67	1101	Citrus	d	d	d	d	d	d	d
24	Nonanal	18.03	1103	Fatty, waxy	d	nd	nd	nd	d	nd	d
25	2-Phenyl alcohol	18.30	1111	Honey-like	d	nd	nd	nd	d	d	nd
26	(*E,Z*)-2,6-Nonadienal *	20.12	1151	Cucumber-like	d	d	nd	d	d	d	nd
27	(*E*)-2-Nonenal *	20.43	1157	Fatty, green	d	d	nd	nd	d	d	d
28	*cis*-Linalool-3,7-oxide	20.93	1168	Sweet, floral, citrus	d	d	nd	nd	d	d	nd
29	*trans*-Linalool-3,7-oxide	21.18	1173		d	d	nd	nd	d	d	nd
30	Methyl salicylate	21.95	1192	-	d	d	d	d	d	d	d
31	Safranal	22.25	1196	Saffron	d	nd	nd	nd	d	d	nd
32	Decanal	22.45	1200	Penetrating, waxy	d	nd	nd	nd	d	d	nd
33	(*E,E*)-2,4-Nonadienal *	22.93	1215	Fatty, green	d	d	d	nd	d	d	d
34	Geraniol *	24.79	1254	Rose-like	d	d	d	d	d	d	d
35	Geranial	25.55	1267	Citrus	d	nd	nd	d	d	d	nd
36	Trans anethole	26.35	1280	Sweet	nd	d	nd	d	d	nd	nd
37	(*E,Z*)-2,4-Decadienal	26.54	1291	Deep-fried	d	d	nd	d	d	d	nd
38	(*E,E*)-2,4-Decadienal *	29.64	1319	Fatty, fried	d	d	nd	d	d	d	nd
39	β-Damascenone	30.48	1381	Fruity	d	d	nd	nd	d	d	nd
40	(*Z*)-Jasmone	31.01	1400	Floral, sweet, fruity	d	nd	nd	d	d	nd	nd
41	α-Ionone	32.24	1424	Violet-like	d	d	nd	d	d	d	d
42	Geranyl acetone	33.31	1450	Magnolia, green	d	d	nd	d	d	d	d
43	β-Ionone *	34.62	1483	Violet-like	d	d	nd	d	d	d	d
44	Caffeine	43.77	1841	-	d	d	d	d	d	d	d

**Table 2:** SA quantitative results on key aroma compounds of tea infusion; data are expressed in µg/L. Odor thresholds are from reference literature [21,27] or from Leibniz-LSB@TUM Odorant Database [43].

Compound Name	Odor Threshold (µg/L)	Ceylon	Assam	Azores	Darjeeling Castleton	Darjeeling Testa Valley	Kenya	Yunnan
3-Methyl butanal	1.2	37.00	49.12	52.32	25.22	27.45	84.89	17.02
2-Methyl butanal	4.4	45.44	63.12	46.69	29.50	21.22	89.97	18.29
Hexanal	10	45.25	23.04	14.84	15.30	63.49	31.75	16.88
(*Z*)-4-Heptenal	0.06	0.98	0.68	<LOQ	0.20	0.82	0.41	0.47
Phenyl acetaldehyde	6.3	32.73	62.73	29.76	10.77	77.52	38.76	16.11
Linalool	0.6	25.75	9.72	10.19	7.86	54.48	27.24	0.41
(*E,Z*)-2,6-Nonadienal	0.03	0.56	0.39	<LOQ	0.50	0.28	0.14	<LOQ
(*E*)-2-Nonenal	0.4	0.40	0.24	<LOQ	0.02	1.21	0.60	0.15
(*E,E*)-2,4-Nonadienal	0.2	0.29	0.39	0.14	<LOQ	1.46	0.73	0.29
Geraniol	3.2	13.20	1.07	16.38	11.31	24.83	12.41	2.78
(*E,E*)-2,4-Decadienal	0.16	0.51	0.15	<LOQ	2.29	0.21	0.11	<LOQ
β-Damascenone	0.004	0.26	0.29	<LOQ	<LOQ	0.38	0.19	<LOQ
β-Ionone	0.2	2.16	0.78	<LOQ	0.18	3.84	1.92	0.29

**Table 3:** Quantitative results on key taste compounds (^T^) and quality markers (^Q^) of selected infusions. Amounts are expressed in mg/L and obtained by external calibration technique with LC-UV/DAD analysis. Results are provided as mean of three infusions and two analytical replicates. Taste threshold (TT) is expressed in µg/L [23,38].

Compound Name	TT (µg/L)	Ceylon	Assam	Azores	Darjeeling Castleton	Darjeeling Testa Valley	Kenya	Yunnan
Epigallocatechin ^Q^ (EGC)	159	72.38	29.24	16.09	25.27	71.08	38.62	16.61
Catechin ^Q^ (C)	119	10.87	8.05	4.42	7.22	13.03	11.54	9.76
Epicatechin ^Q^ (EC)	270	41.97	19.07	12.99	14.16	25.80	24.90	19.84
Epigallocatechingallate ^T,Q^ (EGCG)	87.0	85.35	24.09	11.88	63.52	177.23	27.18	11.23
Gallocatechingallate ^Q^ (GCG)	179	8.64	5.49	2.84	3.16	4.73	3.54	5.44
Epicatechingallate ^Q^ (ECG)	115	41.40	21.90	7.92	29.68	39.79	20.31	18.80
Catechingallate ^Q^ (CG)	239	5.37	5.18	3.07	5.03	3.93	3.59	2.76
Theaflavin ^Q^ (TF)	9.00	7.55	4.39	2.92	2.25	2.16	4.50	2.33
Theaflavin-3-gallate ^Q^ (TF3)	10.7	8.81	7.77	3.70	3.17	2.89	5.90	3.65
Theaflavin-3’-gallate ^Q^ (TF3’)	10.7	5.28	6.64	3.76	3.47	>LOQ	4.92	3.67
Theaflavin-3,3’-gallate ^Q^ (TF3-3’)	11.3	6.38	7.96	>LOQ	3.40	>LOQ	4.77	3.59
Myricetin-3-o-galactoside ^T^ (M-3-o-gal)	1.3	2.39	>LOQ	0.28	0.42	0.32	1.51	>LOQ
Myricetin-3-o-glucoside ^T^ (M-3-o-gluc)	1.0	4.10	0.24	0.37	0.85	1.02	1.33	>LOQ
Quercetin-3-o-rutinoside ^T^ (Rutin)	0.0009	19.58	6.68	6.39	5.16	6.34	9.55	7.63
Quercetin-3-o-galactoside ^T^ (Q-3-o-gal)	0.20	4.31	2.62	1.60	1.90	2.39	4.01	1.82
Quercetin-3-o-glucoside ^T^ (Q-3-o-gluc)	0.30	9.42	3.93	1.01	0.77	1.12	5.53	2.68
Kaempferol-3-o-rutinoside ^T^ (K-3-o-rut)	0.15	8.31	3.11	4.56	1.98	3.83	6.89	3.91
Kaempferol-3-o-glucoside ^T^ (K-3-o-gluc)	0.30	4.19	1.32	1.07	0.62	1.24	3.50	1.43
Caffeine ^T^	97.1	250.50	290.30	143.70	241.52	265.88	263.36	228.03

## Data Availability

Data will be made available on request.

## Supplementary Materials

The following supporting information can be downloaded at https://www.mdpi.com/article/10.3390/molecules29030565/s1, Supplementary Table S1 ST1: Analytes subjected to validation for AI flavor blueprinting of tea. Analytes are listed together with chromatographic information on retention times (*t_R_* min), Target/Qualifier Ions (*m*/*z)* (GC-MS), detection wavelengths (nm) (LC-UV/DAD), calibration functions and determination coefficients (*R*^2^), precision (repeatability and intermediate precision) (CV%), and accuracy results from spiked QC infusions at two levels expressed as percent recovery (% Rec). Accuracy was tested at +10 and +25 µg/L for aroma compounds and +10 and +25 mg/L for tastants and quality markers.
