# Supplementary material for: Artificial Intelligence Sensing: Effective Flavor Blueprinting of Tea Infusions for a Quality Control Perspective
Source: Molecules. 2024 Jan 23;29(3):565. doi: 10.3390/molecules29030565 (PMC10856620; doi:10.3390/molecules29030565)
Supplement: Supplementary file 1 [file molecules-29-00565-s001.zip › molecules-2824860-supplementary.pdf]

The supplementary material comprises one Supplementary Table S1:

Supplementary Table 1 S1: Analytes subjected to validation for AI flavor blueprinting of tea. Analytes are listed together with chromatographic information on retention times (t_R min), Target/Qualifier Ions *m/z* (GC-MS), detection wavelengths nm (LC-UV/DAD), calibration functions and determination coefficients (R^2), precision (repeatability and intermediate precision) CV%, and accuracy results from spiked QC infusions at two levels expressed as percent recovery % Rec. Accuracy was tested at +10 and +25 μg/L for aroma compounds and +10 and +25 mg/L for tastants and quality markers.

Compound name	t_R (min)	Target Ion (m/z)	Qualifiers (m/z)	Detection wavelength (nm)	Intra-week CV%	Inter-week CV%	Calibration function	Linearity (R^2)	Accuracy % Lv 1	Accuracy % Lv 2
(*E*)-2-Nonenal	20.59	55	70, 83, 96		6.94	15.22	y=0.097x+0.35	0.9986	113	116
(*E*,*E*)-2,4-Decadienal	27.92	81	41, 55, 67		4.30	16.56	y=0.44x+1.603	0.9976	109	112
(*E*,*E*)-2,4-Nonadienal	23.22	81	41, 67, 138		9.85	15.87	y=0.429x+0.533	0.9967	115	109
(*E*,*Z*)-2,6-Nonadienal	20.23	41	70		9.97	12.21	y=0.191x+0.867	0.9992	109	106
(*Z*)-4-Heptenal	8.56	41	55, 68, 84		10.63	17.32	y=0.059x+0.468	0.9959	103	100
Beta damascenone	30.49	69	121, 105, 41		7.94	15.40	y=0.37x+0.256	0.9968	100	104
Beta ionone	34.68	177	43, 135		10.86	16.93	y=0.55x+0.14	0.9959	103	110
Butanal, 2-methyl	2.70	57	41, 71, 86		12.56	22.13	y=0.57x+0.23	0.9950	90.0	98.3
Butanal, 3-methyl	2.61	44	58, 71, 86		14.65	24.56	y=0.008x+1.157	0.9972	88.3	94.2
Geraniol	24.82	69	41, 123		9.12	14.25	y=0.14x+5.723	0.9983	102	105
Hexanal	5.21	56	56, 72, 82		8.02	10.09	y=0.034x+9.999	0.9994	114	117
Linalool	17.71	71	93, 121, 55		5.07	14.10	y=0.121x+15.84	0.9969	115	119
Phenyl acetaldehyde	14.96	91	120, 65		11.08	13.54	y=0.127x+50.42	0.9951	106	109
Epigallocatechin	2.50			280	11.96	14.34	y=4833.9x	0.9990	108	107
Catechin	2.85			280	5.91	11.74	y=15840x	0.9988	101	100
Epicatechin	3.45			280	7.56	15.76	y=16605x	0.9989	104	102
Epigallocatechingallate	4.38			280	2.34	14.68	y=29412x	0.9988	108	105
Gallocatechingallate	4.92			280	8.50	11.84	y=36797x	0.9987	107	105
Epicatechingallate	5.50			280	11.22	17.55	y=42214x	0.9986	101	98.9
Catechingallate	6.14			280	4.16	15.97	y=43329x	0.9987	105	104
Theaflavin	7.15			380	2.95	5.10	y=20085x	0.9982	110	108
Theaflavin-3-gallate	7.92			380	6.66	4.98	y=10673x	0.9978	112	109
Theaflavin-3'-gallate	7.59			380	3.23	6.58	y=7408.5x	0.9975	108	106
Theaflavin-3,3'-gallate	8.03			380	2.84	3.99	y=16271x	0.9984	109	103
Myricetin-3-o-galactoside	13.12			350	3.22	9.08	y=31659x	0.9978	99.4	99
Myricetin-3-o-glucoside	13.37			350	13.60	11.07	y=29755x	0.9984	98.6	100
Quercetin-3-o-rutinoside (Rutin)	13.80			350	9.44	14.00	y=34683x	0.9978	96.0	98
Quercetin-3-o-galactoside	14.56			350	4.05	9.79	y=29875x	0.9993	96.9	99
Quercetin-3-o-glucoside	14.87			350	10.01	11.86	y=27784x	0.9968	98.7	102
Kaempferol-3-o-rutinoside	15.40			350	7.08	9.54	y=40086x	0.9982	100	100
Kaempferol-3-o-glucoside	15.79			350	5.84	8.94	y=31976x	0.9968	101	104
Caffeine	12.66			280	5.86	6.00	y=87563x	0.9991	99.0	102
